# Two Cases in Which Turning Could Not Be Accomplished, in Consequence of the Head of the Child Remaining Firmly Fixed at the Brim of the Pelvis, Preventing the Breech Coming down

**Published:** 1855-04

**Authors:** James Wilson


*Two Cases in which Turning could not be accomplished, in consequence of the Head of the Child remaining firmly fixed at the brim of the Pelvis, preventing the Breech coming down.* By JAMES WILSON, M.D. —On May 6th, 1854, the Clerk of the Glasgow Lying-in Hospital was requested by Mr. Hill to visit Mrs. ——, aged 33, residing in St. Ninian Street, Gorbals. Has had several children, all of which were still-born, and more or less premature, two having presented with the feet. All her labors were tedious and difficult. Had arrived at the full period of utero-gestation, and had been in labor eight hours when first visited. The liquor amnii was nearly all discharged, the uterine contractions were powerful and regular, and the os uteri dilated nearly to its full extent. The head was found to present with a loop of the cord prolapsed before it, and pulsating strongly. The sacro-pubic diameter appeared considerably diminished, and the outlet much contracted. The patient, ever since her first delivery, had been strongly impressed with the idea that there existed some pelvic malformation, and stated her belief that she could never give birth to a full-grown living child. She consequently expressed much anxiety and alarm as to her state.

It became a question, whether turning or delivery by the forceps should be had recourse to. Seeing that the pelvic apertures were so contracted, preference was given, with the concurrence of Dr. Stewart, to delivery by turning. As the patient decidedly objected to the inhalation of chloroform, several large doses of laudanum were administered at intervals, with a view to moderate or suspend the action of the uterus, which, however, failed to produce the effect desired. Unwilling to delay any longer, and as Dr. Stewart had been obliged to leave, he (the Clerk) introduced his left hand up to the fundus uteri, seized hold of and brought down the left foot of the child into the vagina. On attempting extraction, a serious and unlooked-for obstacle to delivery arose. The child's head would neither move nor recede, but remained permanently and immoveably fixed at the pelvic brim, while vigorous efforts were made to push it up or aside, so as to allow room for the transit of the breech. During this time the cord was necessarily exposed to much pressure, and its pulsations had nearly ceased. As this locking or wedging-in of the head and foot, proved for the time being an insurmountable barrier to delivery, my assistance was requested. On my arrival, the state of matters was as just described. After great and continued exertion, I ultimately succeeded in pushing the head out of the way, so as to make space for the descent of the breech. Considerable difficulty was experienced in extracting the head, after the body of the child was born. The infant, a full-grown healthy male, was in a state of irrecoverable asphyxia. The placenta came away in the usual time. The mother gradually sunk, and expired 38 hours after the operation, apparently from exhaustion, and excitement produced by officious parties improperly interfering with the necessary operations of the medical attendants, whereby the opportunity was lost for easy and safe delivery.

On the 31st July last, I was called to assist a friend with a case in some respects exactly similar to the one just described. The lady had given birth to one child, a boy, about four hours before, and the doctor was then engaged with the delivery of a second, which he had partly turned. One foot was in the vagina, and also an arm, and the head of the child, as in the above case, was firmly fixed at the brim of the pelvis. Considerable force had been used to bring down the body, but without effect—the head presenting an insurmountable obstacle; and although great pressure had been used to raise it, and remove it out of the way, it remained firmly fixed. I made an attempt to press up the head, and at the same time pulled gently by the foot, without effect. The child was evidently dead. The uterus was acting so powerfully, that we feared rupture would take place, and without further hesitation we decided on perforating the head. We had neither perforator nor crotchet at hand, and, as the case was urgent, I, without difficulty, opened the head, and broke down the brain with a pair of large scissors, and passing my finger into the cranial opening, it answered the purpose of a crotchet very well, at the same time pulling by the protruded arm, the delivery was accomplished without much difficulty. No doubt, the extraction was rendered comparatively easy, in consequence of the powerful action of the uterus. The patient's recovery was perfect.

In these cases there cannot be a doubt that the head remaining at the brim of the pelvis, when the body of the child was brought down, and pressing upon it, was the cause of the difficulty, and also occasioned the children's death. Perhaps the only way to prevent this occurrence in such cases, when version is rendered necessary, is to have recourse to that operation (when we have it in our power) before the uterus is contracted and the waters discharged. When the uterus is uncontracted, and the waters not drained away, the elasticity of the child will move the head from the brim of the pelvis as we bring the feet down; but if the uterus is contracted, and the elasticity of the child gone by death, then the head will remain as in the above cases. When I have had the management of turning from the beginning, this difficulty has never happened to me. I either push the head aside at once, or, when bringing down the foot, I have no difficulty in pressing the head aside with the upper part of the palm or wrist of the same hand with which I hold the foot. Dr. Rigby says, in reference to such cases as the above, "We should perform this step of the operation so gradually, as to be assured that the presenting part has quitted the pelvis before the feet have entered. Without attention to this point, the child may easily be fixed across the upper part of the pelvis, or even the body brought down, while the head is wedged in the *cavitas iliaca* of the ilium, and produce a serious obstacle to its further advance. This sort of mishap can rarely happen, except to young practitioners."* Smellie appears to have guarded against this ever occurring, for in almost all his recorded cases he seems to have turned the child before he brought down the feet. With respect to the first case, it may be said that the delivery should have been effected by perforating the head, as was done in the last case. But as there was no positive proof of the child being dead, it was considered proper to act as I did, with the view of saving the child. I have delivered many children alive, when I experienced greater difficulty than in that case.

I may remark, in conclusion, that in many of those cases where I have been necessitated to perform craniotomy, and where there was no urgent necessity for speedy delivery, I have found it much better, after perforating the head, to permit the woman to rest for a number of hours, whereby the brain becomes completely discharged, the skull collapses, the uterus by rest regains its power, and the child may be expelled by the uterine efforts alone; or at least much less extracting force may be required than had we attempted to complete the delivery immediately after opening the head. For it must be confessed, that though the operation of craniotomy is much easier, and requires less skill than the right use of the forceps, yet the extraction of the child is often attended with the most extreme difficulty to the practitioner and danger to the mother, in consequence of the very great force we are often under the necessity of using.—*Glasgow Medical Journal.*

* Rigby's System of Midwifery, p. 151.

